# Early predictors for late hospitalizations up to 14 years after first episode psychosis

**DOI:** 10.1007/s00127-020-01991-w

**Published:** 2020-12-02

**Authors:** Pontus Strålin, Maria Skott, Johan Cullberg

**Affiliations:** 1 Department of Clinical Neuroscience, Karolinska Institute, Stockholm, Sweden; 2 Department of Medicine, Karolinska Institute, Stockholm, Sweden

**Keywords:** Psychosis, Schizophrenia, Antipsychotic medication, Relapses, Early intervention

## Abstract

**Purpose:**

New hospitalizations after first episode psychosis (FEP) may be viewed as an indicator of instability in a psychotic disorder. In the current study we wanted to analyse long term risk for psychosis hospitalizations after FEP. We also wanted to analyse predictors for late hospitalizations, with focus on early antipsychotic medication.

**Methods:**

First episode psychosis cases were recruited to the Swedish Parachute project in 1996–1997. The program offered highly available and continuous psychosocial support and a cautious use of antipsychotic medication for 5 years from inclusion. Longitudinal data from population registers on psychiatric hospitalizations up to 14 years after inclusion were analysed. One hundred and sixty-one cases were included of the original 175 in the project. Associations with possible early predictive factors from the original project data were analysed with COX regression.

**Results:**

A majority of the cases (67%) had hospitalizations in the first year after inclusion in the study. The cohort then diverged into a group (46%) with new hospitalizations for psychosis after the first year, most of them multiple times, and another group (54%) without new hospitalizations for psychosis, many without any late antipsychotic medication. Forty-two percentage of the cases had antipsychotic medication by month 12, and it was significantly associated with later psychosis hospitalizations (HR = 2.5, *p* value < 0.001).

**Conclusions:**

The study demonstrates that a large part of FEP cases have a good outcome as measured by absence of new hospitalizations for psychosis, and that many cases may terminate antipsychotic medication within a year of FEP onset without later relapses needing hospitalizations.

## Introduction

First episode psychosis (FEP) has a heterogeneous outcome [1–5]. Many cases develop severely debilitating disorders even with continuous antipsychotic medication, while a smaller group may discontinue antipsychotic medication after full remission without relapses [6–12]. In a large meta-analysis of outcome after FEP and with outcome data for more than 1 year, the proportion of cases with remission after FEP was 58% and with recovery 38% [5]. In a study of outcome 10 years after FEP in the OPUS project, 30% of the cases were in symptomatic remission from psychotic symptoms with current antipsychotic medication, and 30% were in remission without current antipsychotic medication [10]. In the 10th year, 17% of the cases had any psychiatric hospitalization [13].

Many factors affect thresholds for psychiatric hospitalizations such as availability of hospitalization services, quality of outpatient care, and individual preferences, and the indications for hospitalizations may vary between countries or regions. Even so, it is reasonable to consider psychiatric hospitalizations in the years after FEP as an indicator of remaining vulnerability and instability in psychiatric states [14]. There are some studies that have investigated the risk for rehospitalization after discharge from a first hospitalization for psychosis. Long term studies have found rehospitalization rates of up to 80% in up to 20 years [15–17]. Several predictors for rehospitalizations have been described including early age at onset [15], a low educational background [18], a short first hospitalization [18, 19], and discontinuation of medication after discharge [16–18].

The Parachute project was based on a 5-year program for early intervention in first episode psychosis. The program offered high availability for social and psychological support for patients and relatives as well as psychoeducational components, delivered in small multidisciplinary FEP teams [20], in a similar fashion to other FEP programs [21, 22]. In contrast to the protocols in many other programs, the policy for early antipsychotic medication was to use it cautiously, principally for positive symptoms with low doses, based on research on D2 dopamine receptor occupancy [23], and not longer than needed to achieve a remission of psychotic symptoms, and minimize side effects.

The overall aim of the current study was to investigate long-term stability of psychotic disorder in the Parachute cohort, as measured by hospitalizations recorded in population registers, after the first year of treatment. In contrast to previous population-based register studies of rehospitalizations after a first hospitalization for FEP, we investigated new hospitalizations for psychosis after the first year of care. The rationale for starting the second year was that many cases have repeated hospitalizations in the often chaotic period around the onset of the FEP, followed by later stabilization in psychiatric states to various extents, and a lower risk for hospitalizations from the second year onwards [12, 24]. The cohort also included cases without hospitalizations in the first year why it is not a focus on “rehospitalizations” as in the previous population register based studies.

The following research questions were investigated: first, what is the rate and volume of hospitalization during a long-term follow-up spanning years 2–14 after initial diagnosis; second, what are early predictors of hospitalizations in the long-term follow-up and third, to what extent does the early use of antipsychotic medication predict time to hospitalization?

## Methods

### Study design and patient recruitment

The current paper presents an exploratory, naturalistic long-term follow-up study of a cohort of FEP cases from the Parachute project. The Parachute project has previously been described in detail [20]. The general principles of the program included interventions for all new FEP patients without delay, with immediate and recurrent family meetings together with the patient, and high accessibility to the multi-professional FEP team during a period of 5 years.

In 1994 the psychiatric clinics in Sweden were invited to participate in the project. After evaluation of interested clinics, 17 clinics throughout Sweden were included to participate, representing a catchment area of about 1.5 million inhabitants, or about one-sixth of Sweden’s population. All clinics were publically financed and had responsibility for the psychiatric care in defined geographic areas. As no private care for early psychosis patients was available in Sweden, we consider the cohort to be rather complete regarding treated incidence of FEP. All the participating clinics offered regular psychiatric inpatient care. In addition, ten of the clinics also offered access to crisis homes as a complement to standard inpatient care. The crisis homes were small scale, homelike, low stimulus overnight facilities with low thresholds for admittance. Patients were usually admitted for short stays when they considered they needed the service.

The collection of cases started 1 January 1996 and ended 31 December 1997 (24 months).

The inclusion procedure and criteria were as follows: patients living in the catchment area, for the first time seeking psychiatric help for psychotic symptoms, age 18–45 years, and without a dominating substance abuse or a diagnosed brain disorder were identified as candidates. During the first week every candidate patient was diagnosed with a SCID interview (axis 1) according to DSM-IV—usually performed by a responsible consultant psychiatrist. Cases with a diagnosis of schizophrenia, schizophreniform psychosis, schizoaffective psychosis, delusional disorder, brief psychosis, psychotic disorder not otherwise specified, or affective disorder with non-congruent psychosis were offered the opportunity to participate in the study.

Two hundred and fifty-three patients were considered to fulfil the criteria for FEP. Based on an estimation of expected incidence of schizophrenia of 0.97 per 10,000 person-years in a general population [25], the expected number of new schizophrenia cases in the catchment area and time interval of inclusion in the study would be around 290. Since the inclusion criteria excluded some cases due to age or other medical conditions, but included a broader set of psychotic disorders, we believe the number of detected cases corresponds roughly to expectations. Of the 253 cases, 175 accepted enrolment at baseline in the study. Cases declining participation were found to have a higher age at FEP and fewer had DSM-IV 295 diagnoses, including schizophrenia [20].

Ten of the 175 patients were recorded as dead prior to 2011. Complete records until 2011 in the population registries were retrieved for 161 cases. For four cases complete records were not retrievable, likely due to emigration. In the current study, the 161 cases were described and analysed. A flow chart of the number of cases in different stages of the process has previously been published [12].

The program had a policy of using the lowest effective doses of antipsychotic medication “as needed” based on symptom severity and clinical judgement. Maintenance medication for the purpose of preventing relapse after remission of positive symptoms was not encouraged. Antipsychotic polypharmacy was generally avoided. Attempts were made to avoid antipsychotic medication during the first 1–2 weeks if possible. Benzodiazepines were used for anxiety or insomnia during this period. Types and doses of antipsychotic medication were chosen based on clinical judgement. All patients were offered psychodynamically oriented psychotherapeutic support during the entire project stay. The method aimed at creating a trustful alliance, supporting cases to verbalize problems, supporting sound self-confidence, confronting stigma, and help patients understand stressful events or feelings. The method was trained within the teams and at regular conferences for the participating clinics. After the 5-year project time, with access to the FEP team, patients needing continuous care were referred to general psychiatric outpatient services, often with less accessibility to support.

### Data collection and follow-up in the first year

All patients underwent thorough psychiatric and medical examinations, including a checklist of background variables at the inclusion. The checklist included data on the highest educational achievement and an assessment of the highest GAF score in the year before onset of FEP, which were used in the current analyses. Data collections were carried out at 5 time points in the first year and after year 3 and year 5 after inclusion, with checklists of current situation and rating scales. In the current study, the following data from the month 12 assessments were used: antipsychotic medication in the 2 weeks preceding the assessment, current symptom ratings with BPRS, used to assess symptomatic remission, and a retrospective SCID-I based diagnosis for the first psychotic episode. The data collection has been described in detail previously [12, 20]. Month 12 assessments were carried out on 158 of the 161 cases included in the current study.

Remission from psychotic symptoms was estimated based on BPRS assessments. The BPRS based criteria proposed by Andreasen et al. [26], but without the prerequisite of 6 months stable state, were used. The variable thus represents a momentary state of remission from psychotic symptoms at the time of assessment.

### Data from population registers

The Inpatient Care Diagnoses Database from the Swedish National Board of Health and Welfare includes all individuals admitted to psychiatric or general hospitals [27, 28] with dates for admission and discharge and ICD-9 diagnoses until 1996, or ICD-10 diagnoses from 1997 for the inpatient care episodes. Hospitalizations for psychosis were identified by ICD-10 F20-F29 diagnoses.

Seven of ten small-scale crisis-homes managed by clinics in the study, were classified as inpatient care and delivered data on admissions, discharges, and diagnoses to the national registries. A few cases thus may have had care in crisis-homes, not recorded in the registers.

The Causes of Death Database comprises information on all deaths of Swedish residents [29].

The Swedish Prescribed Drug Database comprises information on all dispensed prescriptions in Sweden from 1 of July 2005 and onwards [30]. However, it does not cover drugs administered at hospitals.

For analyses of antipsychotics, ACT codes N05A were used, with the exceptions of N05AN (lithium), N05AA02 (levomepromazine), N05AD03 (melperone), and N05AF03 (chlorprothixene) since these medications are usually used for other indications than psychotic symptoms.

Amounts of dispensed medication are reported in the register as number of “defined daily doses” (DDD) for every dispensation. Definitions of one DDD according to the WHO ATC/DDD index [31], for some common antipsychotics are as follows: clozapine 300 mg, olanzapine 10 mg, risperidone 5 mg (2.7 mg for depot), haloperidol 8 mg (3.3 mg for depot), aripiprazole 15 mg, quetiapine 400 mg, paliperidone 6 mg (2.5 for depot).

The longitudinal integration database for health insurance and labor market studies (LISA) from Statistics Sweden includes data on income from employment, payments from sickness benefits and of student aides. Data on income from employment were available for the period from the year of inclusion in the study until year 14 after.

### Statistical methods

For the outcome analyses, the cohort was dichotomized into cases with and without any hospitalizations for psychosis in the years 2–14 after FEP.

Descriptive analyses, Wilcoxon signed-rank tests, odds ratios (OR), Cox regression analysis and Kaplan–Meier survival graphs were made with the R software [32]. Significance levels for odds ratios were calculated based on Wald test statistics.

The counting of cases with hospitalizations in a certain period included all cases with days in hospital in that period. An episode of hospitalization could then be counted several times if it spanned several counted periods.

For the Cox regression analysis of psychosis hospitalization outcome, eight dichotomized factors were used as indicated in Table 2. The data for the factors were derived from the original data collection in the project, except for the data on hospitalizations of the first year, which were derived from register data. The cut-off levels for dichotomization of age was decided based on a trade-off between getting large enough groups of cases, and getting a group of cases as young as possible. The cut off for maximal GAF was based on the standard level for clinically significant symptom severity [33]. The cut-off for income from employment was decided at a relatively low yearly level to include cases with part time work or work a part of the year.

Stepwise forward multivariable Cox regression was performed as follows: first a univariable analysis of all the eight factors was performed. In a first multivariable step, seven factors with *p* value < 0.1 in the univariable analysis were analysed. Stepwise modelling based on the Akaike information criterion (AIC) resulted in the second 4 factor model which was deemed optimal.

Cases with missing data on a variable were excluded from analyses involving the variable.

## Results

### Description of the cohort

Demographic data for the cohort have previously been described [20, 34]. 55% of the cases were males. Mean age at inclusion was 28.7 years. Ten cases had died before 2010 and were not included in the late outcome analyses. Causes of death were suicide in five cases, overdoses of drugs, (unclear if suicidal intentions), myocardial infarctions, and pulmonary embolism in the other cases.

### Primary diagnoses in the cohort

Retrospective SCID-1 based diagnoses for the first psychotic episode were recorded 12 months after inclusion for 153 cases, and missing for eight cases.

The distribution of retrospective diagnoses for the 153 patients were as follows:

- Forty cases (26%) with schizophrenia (DSM-IV 295.1/.2/.3/.6/.9).
- Thirteen cases (8%) with schizophreniform disorder (DSM-IV 295.4).
- Eleven cases (7%) with schizoaffective disorder (DSM-IV 295.7).
- Seventeen cases (11%) with delusional disorder (DSM-IV 297.1).
- Twenty-one cases (14%) with brief psychosis (DSM-IV 298.8).
- Thirty cases (20%) with psychosis NOS (DSM-IV 298.9).
- Twelve cases (8%) with manic of bipolar disorder with non-congruent psychotic symptoms (DSM-IV 296.0/4/5/6/7/8).
- Nine cases (6%) with major depression with non-congruent psychotic symptoms (DMS-IV 296.2/3).

Twenty-nine additional cases were given a schizophrenia, schizophreniform, or schizoaffective diagnosis (ICD10 F20 or F25) for the first time at in-patient or out-patient care in the years 2–14 (based on population registers). In total 93 cases (58%, of the 161 cases in the study population) were given a schizophrenia, schizophreniform, or schizoaffective diagnosis at in-patient or out-patient care within 14 years of follow up. Cases with a first time schizophrenia related diagnosis later than by month 12 were not included in the schizophrenia category in the analyses of outcome prediction.

### Hospitalizations in the first year

Of the 161 cases included in the study, 106 (67%) had any in-patient care in the first year. Fifty cases (31%) had more than one hospitalization in the first year.

The proportion of the study population with hospitalizations declined over time already during the first year. Of the 106 cases, 89 (55%) had any in-patient care in the first month after inclusion, 57 (35%) in the second or third months after, and 42(26%) in the 4th–12th months after (some individuals counted in several time-intervals).

The median number of days of in-patient care in the first year for cases with any in patient care was 29 days with 25% and 75% quartiles at 13 and 76 days.

The median age at inclusion for cases with in-patient care the first year was 29 years compared to 27 years for cases without in-patient care. The difference was not statistically significant (*p* value = 0.32 with Wilcoxon signed-rank test).

Fifty-one percentage of the cases with any psychiatric hospitalization in the first year were women compared to 39% of the cases without. The difference was not statistically significant (OR = 1.37, *p* value = 0.10).

Twenty-six percentage of the cases with in-patient care the first year did not have a high school exam compared to 15% of the cases without in-patient care. The difference was not statistically significant (OR = 1.88, *p* value = 0.15).

### Hospitalizations in the years 2–14

**Fig. 1:**
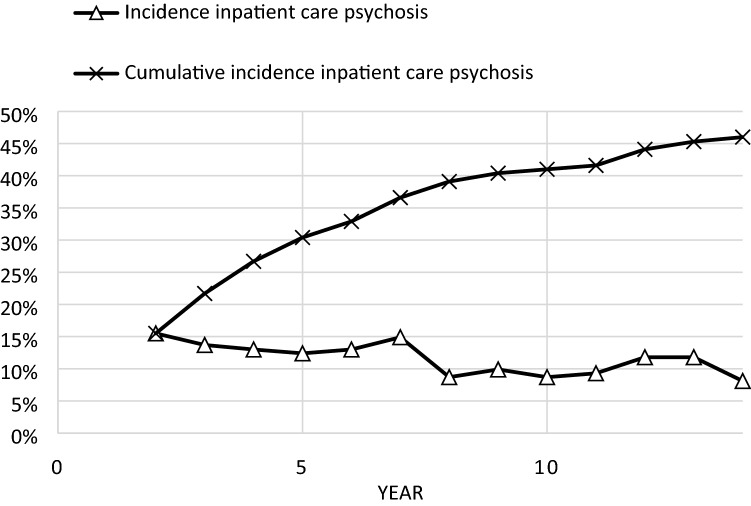
Incidence of hospitalizations for psychosis in the cohort year by year from year 2 after inclusion in the study. Cumulative incidence from the start of the second year and onwards is also presented

There was a continuing trend over time towards lower probability of psychosis hospitalizations. The incidence of hospitalizations for psychosis declined over the years from 15% in year 2 to 8% in year 14 (Fig. 1).

The cumulative incidence of hospitalizations for psychosis in the years 2–14 was 46% (Fig. 1). For cases with any psychiatric hospitalization in the first year after FEP the proportion was 53% (56 of 106 cases).

Of the 74 cases with any psychosis hospitalization in the years 2–14, 19 cases had one episode of hospitalization, nine cases 2 episodes, and 46 cases 3 or more episodes. The median number of days in in-patient care for psychosis during the period, was 79 days with 25% and 75% quartiles at 28 and 270 days.

Seventeen cases (11% of the cohort) had hospitalizations for only other psychiatric disorders than primary psychotic disorders. The most common diagnoses in those cases were manic episodes or bipolar syndrome (ICD10 F30/ F31) in ten cases, and unipolar depression in three cases (F32/ F33). Seven of the cases with affective diagnoses also had specifiers for psychotic symptoms.

We investigated if there was any association between hospitalizations and work record in the late period, and found a strong association between no hospitalizations for psychosis in the years 2–14 and work record in the same period. Fifty-three of the 88 cases (86%) without any hospitalization for psychosis in the period had incomes from regular work higher than 100,000 Swedish kronor (around 10,000 euros) per year for more than 3 years in the period, compared to 20 of 73 cases (27%) with any hospitalization for psychosis in the period. This difference was highly significant (OR = 3.7, *p* value < 0.001).

### Predictors of late hospitalizations for psychosis

**Fig. 2:**
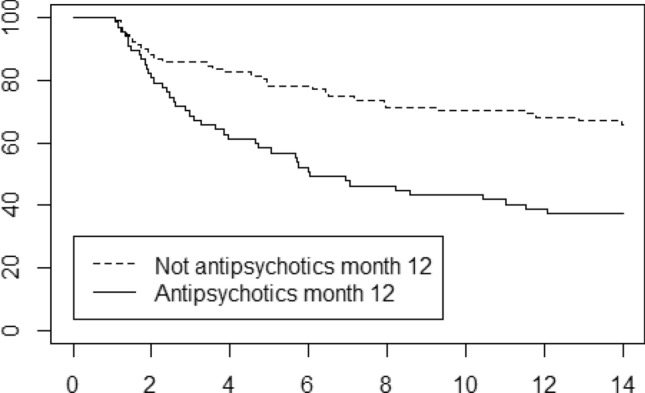
Survival plot of time to hospitalizations for psychosis. Survival counts were started 12 months after inclusion in the study. Cases with and without a prescription of antipsychotic medication month 12 are presented. The *x*-axis indicate years after inclusion in the study. The *y*-axis % of the cohort

Eight possible predictors for psychosis hospitalizations in the years 2–14 were tested which can be seen in Tables 1 and 2. In the first univariable Cox regression analysis the following factors had significant associations (*p* value < 0.05): not having a high school degree, a prescription of antipsychotic medication by month 12 (also Fig. 2), age below 25 years at inclusion, a schizophrenia-related diagnosis (DSM-IV 295) by month 12 and having had in-patient care in the first year after inclusion. Gender did not influence the risk for late hospitalization.

**Table 1:** Distribution of background factors in the cohort and in the groups with and without any hospitalization for psychosis in the years 2–14 after inclusion

	Cohort, *n* = 161	No hospitalization for psychosis years 2–14, *n* = 88	Any hospitalization for psychosis years 2–14, *n* = 73
Female gender	47% (76 of 161)	48%, (42 of 88)	47%, (34 of 73)
Age less than 25 years	37% (59 of 161)	27%, (24 of 88)	48%, (35 of 73)
Not high school exam	22% (35 of 157)	13%, (11 of 86)	34%, (24 of 71)
Maximal GAF the year before inclusion < 70	47% (74 of 156)	40%, (34 of 85)	56%, (40 of 71)
Antipsychotics by month 12	42% (67 of 158)	29%, (25 of 86)	58%, (42 of 72)
Not remission by month 12	32% (47 of 148)	26%, (21 of 82)	39%, (26 of 66)
Schizophrenia-related diagnosis by year 1	40% (64 of 161)	31%, (27 of 88)	51%, (37 of 73)
Any in-patient care the first year	66% (106 of 161)	58%, (51 of 88)	75%, (55 of 73)
Any antipsychotics in the years 11–14	59% (95 of 160)	38%, (33 of 88)	86%, (63 of 73)
Employment more than 3 years in the years 2–14	45% (73 of 161)	60%, (53 of 88)	27%, (20 of 73)

Missing values for some factors are indicated by the number of cases counted for a factor. Schizophrenia-related diagnosis refers to retrospective assessments by month 12. Income from employment of more than 100,000 kr per year was used as a marker for employment

**Table 2:** Cox proportional hazards regression of potential risk factors for psychosis hospitalizations years 2–14

	Univariable		First multivariable		Second multivariable	
	HR	*p* value	HR	*p* value	HR	*p* value
Female gender	0.91	0.6834				
Age less than 25 years	1.90	0.0063	1.86	0.021	1.98	0.0093
Not high school exam	2.28	0.0010	1.99	0.017	1.93	0.0207
Maximal GAF the year before inclusion < 70	1.51	0.0849	1.10	0.73		
Antipsychotics by month 12	2.49	0.0003	2.47	0.0016	2.80	0.0001
Not remission by month 12	1.60	0.0617	1.24	0.44		
Schizophrenia-related diagnosis by year 1	1.84	0.0090	1.19	0.53		
Any in-patient care the first year	1.97	0.0127	1.91	0.030	1.94	0.0257

The multivariable analyses were made on 140 cases with complete data, and the univariable analyses on the number of cases presented in Table 1 for each variable

In multivariable Cox regression, the following factors with *p* values < 0.1 were included in addition to the significant factors mentioned above: “maximal GAF below 70 in the year before inclusion” and “not symptomatic remission by year 1” (Table 2).

The multivariable analysis was carried out stepwise, resulting in a model with the following four factors strongly predicting late in-patient care: a prescription of antipsychotic medication by month 12, not having a high school degree, age below 25 years at inclusion, and having had in-patient care in the first year after inclusion (Table 2).

Descriptive statistics of early antipsychotic medication in the first 5 years in the cohort have previously been published [12]. The median dosage at month 12 was 0.40 DDD/day, and the most common antipsychotic medication was risperidone in 34% of the cases with medication.

### Additional analyses of antipsychotic medication

We did some additional analyses focused on prescription of antipsychotic medication both by month 12 and in the years 11–14 (population register data on medications were not available before year 11, see methods):

There was a strong correlation between antipsychotic medication by month 12 and late antipsychotic medication in the years 11–14. Eighty-three percentage of the cases with a prescription of antipsychotics by month 12 had any dispensing of antipsychotic medication in the years 11–14, compared to 43% of the cases without antipsychotics by month 12. (OR = 6.5, *p* value < 0.001). The median amounts dispensed in the years 11–14 was 273 DDD per year in the group with antipsychotics month 12, and 246 DDD per year in the group without antipsychotics month 12 (only counting cases with late dispensing).

There was a strong correlation between antipsychotic medication by month 12, and a schizophrenia-related diagnosis by month 12. (OR = 4.1, and *p* value < 0.001.)

In the group of cases with a schizophrenia-related diagnosis (DSM-4 295) by month 12, there was a non-significant indication of a difference in risk for later hospitalizations in the years 2–14 between cases with and without antipsychotic medication by month 12. Twenty-six of 40 cases (65%) with antipsychotics by month 12 had later hospitalizations for psychosis, and 11 of 24 cases (46%) without antipsychotics by month 12 (OR = 2.16, *p* value = 0.15).

There was a strong association between any hospitalization for psychosis in the years 2–14 and any antipsychotic medication in the years 11–14. Sixty-two of 72 cases (86%) with hospitalizations had any dispensing of antipsychotics in the period, compared to 33 of 88 cases (38%) without any hospitalization for psychosis in the years 2–14. The different was highly significant (OR = 9.0, *p* value < 0.001).

## Discussion

Based on population register data, we studied outcome after FEP in terms of hospitalizations for psychosis or not after the first year of treatment. We found that around half of the cases had hospitalizations for psychosis after the first year, most of these cases with multiple hospitalizations, and that the other half did not. Cases without any hospitalizations for psychosis in the period we think represent a group with more stable psychiatric states in terms of psychosis symptoms, even though there is room for substantial psychiatric symptoms without needing hospitalizations.

Considering that there is a substantial variation between countries in the proportion of cases that are hospitalized in the years after FEP [15], depending on for example differences in availability of hospital care and of other types of care and support, it is difficult to draw conclusions from differences in proportions of hospitalizations. Several studies from the Nordic countries that have looked at rehospitalizations after a first hospitalization for psychosis have noted higher proportions of rehospitalizations, up to 80% of the cases [17, 18, 35]. Unfortunately the methodology in those studies differed in substantial ways from the current study. There was in all a different selection of the study populations, focusing on schizophrenia, and a selection of cases with a first psychosis hospitalization, while the current study included a wider group of FEP cases, including some that did not have any early hospitalization, likely representing less severe disorders. The time frame for analysing risk for new hospitalizations was from discharge in the studies referred, while the current study started from the second year onwards. We believe that the current outcome measure, starting after a year, gives a better indication of long term stabilization.

In the 10 years outcome study of the Danish OPUS project [13], there was no analysis of cumulative incidence of hospitalizations over many years but presentations of prevalence of hospitalizations in the years 5 and 10. The proportions of hospitalizations were higher in the OPUS study, for year 10, 17% compared to 9% in the Parachute cohort. Though it is not possible to draw definite conclusions from these differences due to likely unspecific differences between the Danish and Swedish settings, the results anyways indicate a good outcome in the current study. Likely the ambitious early intervention program contributed, as has been shown for the OPUS project [36], and maybe also the cautious use of antipsychotic medication.

We found several possible predictors for late hospitalizations for psychosis. As previously described, there was a strong association between a low educational level and late hospitalizations [18]. We believe that this factor likely reflect a low premorbid cognitive level in many cases, even though other explanations may exist in some cases, such as maltreatment or poverty during upbringing. Hospitalizations in the first year after onset was also strongly associated with later hospitalizations, likely reflecting more severe psychotic disorders.

As in previous studies, low age at onset of FEP was associated with later hospitalizations [15]. This factor may reflect more severe psychotic disorders or possibly less cognitive resources for the central nervous system to cope with and recover after FEP, with a higher risk for remaining instability in psychic states.

While gender has been associated with other outcome measures after FEP [34, 37, 38], there was no indication of influence on the late hospitalization for psychosis outcome in the current study. This illustrates the multidimensional character of outcome after FEP, and how different outcome measures may have very different dependencies on premorbid and other risk factors.

We found a strong association between antipsychotic medication by month 12 after FEP and later hospitalizations for psychosis. This finding is in contrast to some previous studies that have found a high risk for new hospitalizations after discontinuation of antipsychotic treatment after FEP [17, 18, 39, 40]. There may be several factors that contribute to the better outcome after discontinuation of antipsychotic medication in the current study. One factor may be that the current study included a wider spectrum of first episode psychosis cases, where schizophrenia related disorders constituted 40% of the cases and only 66% of the cases had any psychiatric hospitalization in the first year. The wider spectrum likely includes a larger group of cases with less severe psychotic disorders and thus a larger proportion of cases likely not needing long term antipsychotic treatment.

A schizophrenia related diagnosis by month 12 was actually also a predictor for later hospitalizations in the univariable HR analyses in the current study, antipsychotic medication by month 12 correlated with a schizophrenia related diagnosis, but had a stronger correlation than a schizophrenia related diagnosis to late hospitalizations in the univariable analyses. The medication factor likely absorbed the diagnosis factor in the multivariable analysis.

In the Parachute project there was a policy of cautious treatment with antipsychotic medication. The psychiatrists would encourage cases to stop using antipsychotics if they found them recovered from acute symptoms. Another possible explanation for the association between medication month 12 and later hospitalizations is then that in the Parachute project, medication by month 12 reflects severity in the psychotic disorder, and do it with better predictive power than a schizophrenia related diagnosis, at least as measured with later hospitalizations as outcome.

Another hypothetical factor is that long term use of antipsychotic medication may increase the risk for later hospitalizations after termination. Hypotheses around super-sensitivity of dopamine receptors or other slow adaptations of the dopamine system to antipsychotic medication have suggested such a connection [41]. Some epidemiological results also have raised the question of a risk for rebound psychosis after termination of antipsychotic medication. A large study of a Finish cohort noted that around 30% of the cases with early termination of antipsychotics were not re-hospitalized, but lower proportions of cases with later termination [17]. More research is needed to clarify if there is such a connection.

That long term antipsychotic treatment is undoubtedly indispensable for many cases with schizophrenia and other psychotic disorders has been demonstrated not least in large population register based studies [17, 42, 43]. At the same time, the current study among other [10, 12], indicate that there is another large group of FEP cases that will not need long term antipsychotic medication for a good outcome, and that may terminate the treatment within the first year after psychosis onset. Considering the high prevalence of side effects of antipsychotic medication, we believe it is important to find treatment algorithms that limit the long term use to cases that really need it to achieve symptom control [41, 44]. More research is needed to improve methods for predicting which FEP cases may terminate antipsychotic medication early, and which cases need long term treatment.

In conclusion, the study demonstrates that a large part of FEP cases have a good outcome as measured by the absence of new hospitalizations for psychosis, and that many may terminate antipsychotic medication within a year of FEP onset without later relapses needing hospitalizations.

There were several limitations to this study. The sample size was rather small which limited the power for analyses on sub-groups. There was no randomization to different treatment arms for antipsychotic medication, which for example means that the choice of antipsychotic medication at month 12 was dependent partly on uncontrollable factors. The register data on medications was only available from July 2005, which limited the knowledge of the use of antipsychotic medication in the years 2–10 under study.

## Data Availability

Available on demand from the authors.
